# Interaction Between Cigarette Smoking and PARK16 on Parkinson’s Disease

**DOI:** 10.3390/ijms27198556

**Published:** 2026-09-24

**Authors:** Tze Tsung Chong, Jia Dong James Wang, Ebonne Yu-Lin Ng, Qiao-Yang Sun, Eng-King Tan

**Affiliations:** 1 Department of Neurology, National Neuroscience Institute, Singapore General Hospital Campus, Singapore 168581, Singapore; chongtt@u.duke.nus.edu (T.T.C.); jwang082@e.ntu.edu.sg (J.D.J.W.); ebonne.ng.y.l@sgh.com.sg (E.Y.-L.N.); qiaoyangsun001@gmail.com (Q.-Y.S.); 2 Duke-NUS Medical School, Singapore 169857, Singapore; 3 Lee Kong Chian School of Medicine, Nanyang Technological University, Singapore 308232, Singapore

**Keywords:** Parkinson’s disease, gene-environment interaction, smoking, oxidative stress

## Abstract

Parkinson’s disease (PD) is influenced by genetic and environmental factors. Epidemiological evidence indicates that smoking is associated with reduced PD risk, potentially through oxidative stress-related pathways. *PARK16* is a PD-susceptibility locus also implicated in oxidative stress regulation. This study examined gene–environment interaction involving smoking and a *PARK16* single nucleotide polymorphism (SNP). A case–control study involving 999 Chinese subjects (497 cases, 502 controls) was conducted. *PARK16* SNP rs6679073 (NC_000001.11:g.205787356C>A) was identified in a genome-wide association study in an Asian population. Logistic regression estimated adjusted odds ratios (ORs), controlling for age, sex, family history, and comorbidities. Gene–environment interactions were assessed using both additive (RERI, AP, and S) and multiplicative models. Smoking was not significantly associated with PD risk in the cohort (adjusted OR 0.78, 95% CI 0.54–1.12, and *p* = 0.179). rs6679073 was significantly associated with increased PD risk under the recessive model (AA vs. CC/CA: adjusted OR 1.47, 95% CI 1.11–1.93, and *p* = 0.007). An additive interaction between rs6679073 and smoking was observed (RERI 0.85, 95% CI 0.21–1.50; AP 0.51, 95% CI 0.12–0.89). These findings suggest a biologically plausible interaction between *PARK16* rs6679073 and cigarette smoking in modulating PD risk and warrants further investigation. Understanding gene–environment interactions may inform the design of mechanistic studies and contribute to risk stratification in susceptible populations.

## 1. Introduction

Parkinson’s disease (PD) is a progressive neurodegenerative disorder characterised by the classic motor features of tremors, bradykinesia, and rigidity, arising from the loss of dopaminergic neurons in the substantia nigra [1]. In Singapore, PD affects approximately three in every 1000 individuals aged 50 and above [2]. Despite being the second most prevalent neurodegenerative disorder after Alzheimer’s disease, PD is the fastest-growing globally [3]. Although ageing is the primary risk factor, both genetic and environmental factors contribute significantly to disease onset and progression [1,4].

Large-scale genome-wide association studies (GWASs) have identified up to 90 PD-associated genetic variants, accounting for an estimated 16–36% of heritable risk [5,6]. An Asian-specific GWAS also revealed 11 genome-wide significant loci, including two that are novel [7]. Separately, epidemiological studies have highlighted lifestyle factors that modify PD risk, with cigarette smoking consistently showing a paradoxical protective association [1,4,8]. The limited explanatory power of genetic factors alone, combined with the robust evidence for environmental influences, suggests that gene–environment interactions may underlie many cases of sporadic PD [9,10]. Prior studies have reported variable results in the interaction between smoking and PD-associated genes in various ethnic populations [11,12,13,14,15].

Several studies, including those employing Mendelian randomisation, dose–response analyses, and twin-study designs, have suggested a robust protective effect of smoking on PD [16,17,18,19,20]. However, the underlying biological mechanisms remain incompletely understood. Previous studies propose that cigarette constituents such as nicotine and carbon monoxide may have neuroprotective properties and have been shown in preclinical models to reduce neuroinflammation and oxidative stress [21,22,23,24].

*PARK16* is a PD-susceptibility locus on chromosome 1 that encompasses the following five genes with distinct biological functions: *SLC45A3*, *NUCKS1*, *RAB7L1*, *SLC41A1*, and *PM20D1* [25]. It was first identified in a GWAS conducted in a Japanese cohort in 2009 [26], and subsequent studies across diverse ethnic populations have replicated its association with PD, though with notable variability in allele frequencies and effect sizes [25]. Evidence suggests that *PARK16* plays a role in oxidative stress pathways, a key mechanism in PD pathogenesis, and may interact with other genes like *LRRK2*, thereby altering vesicular trafficking function and related cellular processes [27,28,29,30].

Several variants within the *PARK16* locus have been implicated in PD susceptibility, yet their interactions with environmental exposures remain poorly characterised. While a prior Japanese study investigated gene–smoking interaction involving *PARK16* single nucleotide polymorphisms (SNPs) identified in the 2009 Japanese GWAS [13], no such study has been conducted in a southeast Asian cohort where genetic architecture may differ. Given the central role of oxidative stress in PD pathogenesis, and that the *PARK16* SNP is among the top GWAS hits in the Asian population [7], this study aims to investigate gene–environment interaction involving cigarette smoking and *PARK16* SNP rs6679073 using an established PD database from Singapore. Gene–environment interactions offer an important strategy to gain a deeper mechanistic understanding of the smoking-PD association and may contribute to precision medicine approaches by stratifying risk by genotype and exposome and facilitating targeted prevention for high-risk groups [31].

## 2. Results

A total of 999 subjects (497 cases, 502 controls) were included in the cohort. Controls were younger than cases at assessment but had a mean age that exceeded the mean age at onset of cases (controls: 62.10 ± 7.15 years; cases 63.84 ± 6.06 years; and age at onset 60.83 ± 7.82 years) (Table 1). Although this difference was statistically significant, it was small and is unlikely to be clinically meaningful. Compared with controls, cases were more likely to have a positive family history of PD or related movement disorders and they also exhibited a higher prevalence of comorbidities like hypertension and diabetes mellitus. No significant differences were observed between cases and controls with regard to sex distribution or prevalence of hyperlipidaemia. No statistically significant association was observed between smoking and PD risk after adjusting for age, sex, family history, and comorbidities (adjusted odds ratio, OR 0.78, 95% confidence interval, CI 0.54–1.12, and *p* = 0.179).

In the cohort controls, genotype frequency for rs6679073 did not deviate from Hardy–Weinberg equilibrium (*p* = 0.20). The A allele was more prevalent than the C allele, consistent with allele frequency reports in the Asian population from the dbSNP database [32] (Table 2). In the multivariable logistic regression model, rs6679073 was significantly associated with increased PD risk under the recessive model (adjusted OR 1.47, 95% CI 1.11–1.93, and *p* = 0.007). Interestingly, under the co-dominant model (reference genotype: CC), the CA genotype appeared to confer a protective effect, whereas the AA genotype was associated with increased PD risk. However, these associations were not statistically significant.

For gene–environment interaction analysis under the recessive model, never smokers with the rs6679073 AA genotype had a higher PD risk as compared to ever smokers with the CC or CA genotypes (adjusted OR 1.68, 95% CI 1.04–2.70, and *p* = 0.033) (Table 3). A statistically significant additive interaction between smoking and rs6679073 was observed, as indicated by two measures, relative excess risk due to interaction (RERI) and attributable proportion due to interaction (AP) (RERI = 0.85, 95% CI 0.21–1.50; AP = 0.51, 95% CI 0.12–0.89). However, the multiplicative interaction was non-significant (interaction OR 2.04, 95% CI 0.99–4.21, and *p* = 0.054). The synergy index (S) was not estimable. No significant associations were observed under the dominant model. As comorbidities such as hypertension, hyperlipidaemia, and diabetes mellitus may share causal pathways with smoking, potentially acting as mediating factors rather than true confounders, adjustment for these variables may introduce collider bias. Thus, a sensitivity analysis excluding these comorbidities was conducted, with similar results (Supplementary Table S1).

## 3. Discussion

*PARK16* was first identified as a PD-susceptibility locus in a 2009 Japanese GWAS [26]. Subsequently, Tan et al. validated the findings in the Chinese population, reporting that five *PARK16* SNPs had a protective effect on PD (OR range: 0.64–0.84) [33]. The present study found that in a Chinese cohort, rs6679073 showed a statistically significant association with PD under the recessive model, with increased risk of PD among AA carriers. Under the co-dominant model, neither the CA or AA genotype individually reached statistical significance in reference to CC, and the observed pattern of opposing effect directions should therefore be interpreted with caution. Moreover, a statistically significant additive interaction was observed between rs6679073 and smoking, although the multiplicative interaction term was not significant. Nonetheless, the additive model for interaction was prioritised as it more appropriately captures biological interaction between two factors via a departure from additivity in disease risk [34]. These findings suggest a biologically plausible gene–environment interaction between *PARK16* SNP rs6679073 and smoking. This is consistent with prior findings on *PARK16* in a Japanese cohort, which reported an additive interaction for rs823128, albeit with a smaller effect size for RERI but comparable AP values for our study [13]. Nevertheless, this finding should be interpreted with caution due to the lack of a significant multiplicative finding and warrants replication.

*PARK16* SNP rs6679073 is an intergenic variant located between *RAB7L1* and *SLC41A1* and may not be the functional variant responsible for the observed association. It may instead serve as a signal in linkage disequilibrium with other functional variants within the locus or other causal genes. The following discussion addresses plausible mechanisms at the *PARK16* locus more broadly: *SLC41A1* encodes a Na^+^/Mg^2+^ exchanger implicated in magnesium homeostasis, and dysfunction may lead to a magnesium-deficient state and increased oxidative stress [27,28]. RAB7L1 (Rab29) has been implicated in a shared pathway with another PD-susceptibility gene, *LRRK2*, affecting retromer function and vesicular trafficking, processes relevant to PD pathogenesis [30,35]. Another gene further downstream in the *PARK16* locus, *PM20D1*, encodes an enzyme that catalyses the formation of N-arachidonoyl dopamine (NADA), an antioxidant molecule that also reduces α-synuclein aggregation [29].

Among smoking constituents, nicotine is a natural candidate in mediating the smoking-PD association due to its consistent presence in tobacco products and biologically plausible mechanisms [36]. Nicotine may exert its neuroprotective effects particularly via the α7 nicotinic acetylcholine receptor (nAChR), which is widely expressed in the substantia nigra [36,37]. Specifically, preclinical studies have demonstrated that this involves activation of the phosphatidylinositol-3-kinase (PI3K)/Akt pathway, thereby reducing neuroinflammation and oxidative stress, and promoting neuron survival and resistance to excitotoxic insults [24,37]. Nicotine analogues have similarly been reported to reduce oxidative stress in PD models [23], and machine learning approaches have predicted neuroprotective potential for these analogues, also mediated by the PI3K/AKT pathway [38]. Despite this, the largest nicotine clinical trial to date (NIC-PD) evaluating transdermal nicotine therapy in 163 early-stage PD patients found no significant effect on disease progression [39]. This may reflect the possibility that nicotine’s neuroprotective effects are restricted to patient subgroups based on genotype, or that other constituents may drive the observed protective association. CO, despite traditionally being viewed as toxic, has demonstrated anti-inflammatory and antioxidant properties in preclinical models, potentially via upregulation of HO-1 expression [21,22], and may have a neuroprotective role by reducing αSyn pathology [40]. Along with the aforementioned *PARK16* genes, these represent biologically plausible mechanisms that may underlie the observed interaction between *PARK16* and smoking, which involve oxidative stress pathways. However, these interpretations require further investigation and should be interpreted as hypothesis-generating, pending validation through dedicated functional and molecular studies.

This study has several strengths. While *PARK16* is an established PD-susceptibility locus, prior work has largely focused on genetic association, with limited investigation of interactions with environmental exposures. The present study extends existing knowledge by investigating the potential interplay between *PARK16* rs6679073, which was among the top signals of an Asian GWAS, and smoking, a consistently observed environmental modifier of PD risk. This contributes to the growing body of the gene–environment literature and provides the foundation for further studies.

Exposure and covariate data were collected using a prospectively validated protocol with demonstrated repeatability, supporting the reliability of the data used in this analysis. Logistic regression models were adjusted for relevant covariates such as family history and comorbidities, all of which may be confounders in the observed association. However, residual confounding cannot be entirely excluded. Inclusion of comorbidities in the regression model may introduce collider bias if these conditions lie on the causal pathway between smoking and PD. However, a sensitivity analysis conducted showed similar results with the primary analysis, mitigating these concerns. Based on the observed allele frequency of rs6679073 in the recessive model, an assumed smoking prevalence of 20%, and marginal gene and smoking effects of 1.50 and 0.60 respectively, the cohort sample size achieves power > 80% to detect an interaction OR of 2.60. The observed multiplicative interaction OR of 2.04 fell below this threshold, which may explain why statistical significance was not reached despite similar effect direction.

Several limitations should also be acknowledged. Demographic, clinical, and exposure information were self-reported and subject to recall bias and confounders such as cognitive dysfunction in older PD patients, even though this was partially mitigated using the validated protocol. Smoking data were assessed as a binary “ever” vs. “never” classification and lacked detailed quantitative information (e.g., duration or amount), limiting the assessment of dose–response relationships. This distinction could not be reliably derived, as detailed smoking history was not consistently captured during earlier phases of data collection. This precludes assessment of whether the interaction is modified by smoking intensity or cumulative exposure, which would strengthen confidence in the findings. Current and former smokers may also have fundamentally different exposure profiles, and this binary classification would not allow subgroup comparison which may mask heterogeneity in the observed effect. Further, as passive smoking may be associated with reduced PD risk [17], not controlling for this variable among control participants recruited from patient spouses may lead to misclassification bias and distort risk estimates. Exposure data were also captured at one time point, without accounting for potential fluctuations in smoking habits over time.

A further limitation concerns the possibility of cohort overlap between the present study and the Asian GWAS [7], and our study does not constitute an independent replication of the GWAS signal in *PARK16*. The GWAS involved aggregated data across 13 Asian sites, and no gene–environment interaction analysis was carried out, which represents the novel contribution of the present study. Additionally, as only the selected SNP was genotyped rather than whole sequencing, population stratification within the Chinese cohort was not assessed. Residual confounding from population substructure within this ethnic group cannot be entirely excluded. Lastly, adjustment for other variants in PD-susceptibility genes (e.g., *SNCA*, *LRRK2*, and *GBA*) was not performed. As sporadic PD is a polygenic disorder, other variants may confound the observed interaction if these variants in PD risk loci are correlated with the *PARK16* genotype or smoking behaviour. Further studies should thus incorporate genome-wide sequencing or adjustment for polygenic risk scores to determine whether this interaction persists.

Further studies are warranted to replicate these findings in larger sample sizes of other ancestries to determine whether this finding is population-specific. The *PARK16* locus could be investigated in greater detail using fine-mapping methods given the presence of multiple genes with plausible biological mechanisms. A recent cross-ancestry Mendelian randomisation study identified PM20D1 as a causal protein specifically associated with reduced PD risk in east Asians, suggesting that different genes within this locus may drive ancestry-specific disease mechanisms [41]. Future studies may use similar methods, integrating genomic and proteomic data, to help clarify which *PARK16* genes underlie the smoking interaction. Exposure assessment can be improved to enable more thorough analysis such as dose–response relationships. Integrating exposure data with objective biomarkers of smoking exposure such as cotinine or other tobacco-related metabolites [42] can provide greater biological correlation for the exposure variable. Given that smoking has been associated with increased ventral striatum dopaminergic activity observed on positron emission tomography [43], and other studies have used striatal dopamine transporter (DAT) binding and grey matter volume to examine the smoking-PD association [44], such neuroimaging techniques could be used to further corroborate findings. Finally, there is translational potential to elucidate the underlying biological mechanisms using human midbrain organoids or transgenic PD animal models to examine gene–environment effects at a molecular level.

In summary, the study provides evidence supporting a potential biological interaction between *PARK16* and smoking in influencing PD risk. These findings add to the understanding of PD pathogenesis and offer insights into the possible protective mechanisms of smoking, potentially through oxidative stress-related pathways. If validated, these findings may be included in risk-stratification models to identify individuals who are genetically predisposed to PD, but whose risk could be modified by environmental exposures. Such insights may facilitate targeted preventive strategies, including enrolment of high-risk individuals in clinical trials evaluating novel therapeutics involving the implicated pathways. However, we emphasise that any future clinical guidance must avoid implying that smoking represents an acceptable preventive exposure, given its well-established adverse health impacts.

## 4. Materials and Methods

### 4.1. Study Demographics

This study was approved by the SingHealth Centralised Institutional Review Board. A case–control study design was employed, and the dataset utilised a cohort of Chinese PD patients and healthy controls recruited at Singapore General Hospital, National Neuroscience Institute, and SingHealth Group clinics. Eligible cases were diagnosed by a movement disorder specialist in accordance with the UK Parkinson’s Disease Society Brain Bank clinical diagnostic criteria [45]. Prior studies have reported misdiagnosis rates of 5–10% among movement disorder specialists applying standard criteria [46]. To mitigate this risk, PD cases in the present study were longitudinally followed up, and any participant who subsequently developed clinical features fulfilling exclusion criteria was removed from the study. Control participants were drawn from patient spouses and referrals, community outreach activities, and health screening events and had no clinical evidence of Parkinsonism or other movement disorders. They were sex-matched to cases and randomly selected from the database.

All participants completed an in-person questionnaire that captured clinical and demographic information. Data on age, sex, family history of PD or related movement disorders, and smoking history were extracted. Smoking status was categorised as ever-smokers if they were current or former smokers, and never smokers otherwise. Participants with incomplete smoking data were excluded from the analysis.

Clinical data on comorbidities such as hypertension, hyperlipidaemia, and diabetes mellitus were also extracted and included as covariates due to their potential confounding effects. Hypertension and diabetes mellitus have been independently associated with PD risk [1,4,47], including studies done in the Singapore population [48]. Evidence for hyperlipidaemia and PD risk is less consistent, with studies reporting associations in both directions [1,4,49,50], but hyperlipidaemia was retained as a covariate for consistency with the prior study examining vascular risk factors and PD risk in this population [48].

Smoking exposure and clinical data were evaluated using a protocol that had been prospectively validated in the same clinics. This involved a structured questionnaire administered by a clinical research coordinator, containing questions pertaining to smoking status (e.g., Did you ever/do you smoke? Still smoking?), as well as self-reported comorbidities (e.g., Do you have hypertension? Do you suffer from diabetes?). Participants were classified as having a positive family history if they had any first-degree relative with PD or related movement disorders. Information on smoking exposure and comorbidities like hypertension and diabetes mellitus demonstrated robust repeatability over time [51]. A total of 999 subjects (497 cases, 502 controls) were included in the analyses.

### 4.2. Genomic Data

*PARK16* SNP rs6679073 (NC_000001.11:g.205787356C>A) was identified in a GWAS done on the Asian population [7].

Genomic DNA was extracted from 5 to 20 mL of subjects’ venous blood samples using column-based kits manufactured by Qiagen GmbH, Hilden, Germany (QIAamp DNA Blood Maxi kit, QIAamp DNA Blood Midi kit). DNA quality was assessed using A260/A280 ratios which ranged from 1.79 to 1.87 in this cohort. rs6679073 was genotyped using TaqMan SNP assays on the ABI 7500 Real Time PCR System (Applied Biosystems, Thermo Fisher Scientific, Waltham, MA, USA). Genotyping was successful for all 999 subjects. Duplicate samples were not used.

### 4.3. Statistical Analysis

Statistical analyses were conducted using IBM SPSS Statistics for macOS, version 30.0 (IBM Corp., Armonk, NY, USA). Power calculations were performed using QUANTO version 1.2.4 (Keck School of Medicine, University of Southern California, Los Angeles, CA, USA) [52]. Hardy–Weinberg equilibrium was tested in controls using the Pearson chi-square test. Logistic regression analyses were performed to calculate ORs, 95% CIs, and *p*-values (statistical significance set at *p* < 0.05) for PD. The reference group was defined as individuals homozygous for the reference allele, as determined by the National Centre for Biotechnology Information (NCBI) dbSNP database [32]. SNP effects were examined using co-dominant, additive, dominant, and recessive genetic models. Multivariable logistic regression models were used, adjusting for age (continuous variable), sex, family history of PD or related movement disorders, and comorbidities (hypertension, hyperlipidaemia, and diabetes mellitus). Missing data were handled using complete-case analysis as the proportion of missing data was small across both cases and controls (<1%), minimising the risk of bias.

Gene–environment interactions were evaluated using both additive and multiplicative models. Analyses were performed using the dominant and recessive genetic models only, as the method described by Andersson et al. [53] requires each factor to be dichotomised, and to limit the number of statistical comparisons performed. Additive interaction was assessed using the following three measures of biological interaction: RERI, AP and S. A biological interaction is suggested if the RERI or AP values deviate from 0 or if S deviates from 1. CIs were obtained via the delta method, and statistical significance was determined if the 95% CI excludes 0 (RERI and AP) or 1 (S). Multiplicative interaction was assessed by introducing a product term between smoking status and the SNP into the logistic regression model.

## Figures and Tables

**Table 1:** Baseline characteristics of the Chinese cohort.

	Controls (*n* = 502)	Parkinson’s Disease (*n* = 497)	*p*-Value ^1^
Age (years)	62.10 ± 7.15	63.84 ± 6.06	<0.0000376
Age at onset, AAO (years)		60.83 ± 7.82	
No data for AAO (*n*, %)		49 (9.9)	
Sex			
Males (*n*, %)	251 (50.0)	247 (49.7)	0.924
Females (*n*, %)	251 (50.0)	250 (50.3)	
Family history of Parkinson’s disease (PD) or related movement disorders			
Yes (*n*, %)	35 (7.0)	80 (16.1)	0.00000499
No (*n*, %)	467 (93.0)	413 (83.1)	
No data (*n*, %)	0	4 (0.8)	
Smoking history			
Yes (*n*, %)	96 (19.1)	82 (16.5)	0.278
No (*n*, %)	406 (80.9)	415 (83.5)	
Hypertension			
Yes (*n*, %)	140 (27.9)	222 (44.7)	3.39 × 10^−8^
No (*n*, %)	361 (71.9)	274 (55.1)	
No data (*n*, %)	1 (0.2)	1 (0.2)	
Hyperlipidaemia			
Yes (*n*, %)	169 (33.7)	163 (32.8)	0.771
No (*n*, %)	332 (66.1)	333 (67.0)	
No data (*n*, %)	1 (0.2)	1 (0.2)	
Diabetes mellitus			
Yes (*n*, %)	51 (10.2)	99 (19.9)	0.0000157
No (*n*, %)	450 (89.6)	397 (79.9)	
No data (*n*, %)	1 (0.2)	1 (0.2)	

^1^ *p*-values were calculated using the chi-square test for categorical variables and the independent samples *t*-test for continuous variables.

**Table 2:** Association between *PARK16* rs6679073 and PD.

*n* (Controls/Cases)	Model	Genotype	*n* (%)		Crude OR (95% CI)	Adjusted OR (95% CI) ^1^	*p*-Value
			Controls	Cases			
999 (502/497)	Co-dominant	CC	92 (18.3)	97 (19.5)	1.00	1.00	
		CA	262 (52.2)	213 (42.9)	0.77 (0.55–1.08)	0.78 (0.54–1.10)	0.158
		AA	148 (29.5)	187 (37.6)	1.20 (0.84–1.71)	1.22 (0.84–1.78)	0.293
	Additive	Per additional A allele			1.15 (0.96–1.37)	1.16 (0.97–1.40)	0.110
	Dominant	AA + CA vs. CC			0.93 (0.67–1.27)	0.94 (0.67–1.31)	0.698
	Recessive	AA vs. CC + CA			1.44 (1.11–1.88)	1.47 (1.11–1.93)	0.007

^1^ Logistic regression models were adjusted for age, sex, family history, smoking history, hypertension, hyperlipidaemia and diabetes mellitus.

**Table 3:** Interaction between *PARK16* rs6679073 and smoking status on risk of PD.

Genetic Model	Smoking Status	Genotype	*n* (%)		Crude OR (95% CI)	Adjusted OR (95% CI) ^1^	*p*-Value
			Controls	Cases			
Dominant	Yes	CC	16 (3.2)	17 (3.4)	1.00	1.00	
		CA + AA	80 (15.9)	65 (13.1)	0.77 (0.36–1.63)	0.80 (0.36–1.75)	0.573
	No	CC	76 (15.1)	80 (16.1)	0.99 (0.47–2.10)	1.10 (0.50–2.43)	0.816
		CA + AA	330 (65.7)	335 (67.4)	0.96 (0.48–1.92)	1.07 (0.51–2.23)	0.866
Recessive	Yes	CC + CA	63 (12.5)	55 (11.1)	1.00	1.00	
		AA	33 (6.6)	27 (5.4)	0.94 (0.50–1.75)	0.82 (0.42–1.58)	0.547
	No	CC + CA	291 (58.0)	255 (51.3)	1.00 (0.67–1.50)	1.01 (0.65–1.57)	0.972
		AA	115 (22.9)	160 (32.2)	1.59 (1.03–2.46)	1.68 (1.04–2.70) ^2^	0.033

^1^ Logistic regression models were adjusted for age, sex, family history, hypertension, hyperlipidaemia and diabetes mellitus. ^2^ Interaction analyses: relative excess risk due to interaction (RERI) = 0.85 (95% CI 0.21–1.50); attributable proportion due to interaction (AP) = 0.51 (95% CI 0.12–0.89); multiplicative interaction OR 2.04 (95% CI 0.99–4.21, *p* = 0.054). Synergy index (S) was not estimable.

## Data Availability

The data presented in this study are available upon reasonable request from the corresponding author due to IRB restrictions.

## Supplementary Materials

The following supporting information can be downloaded at https://www.mdpi.com/article/10.3390/ijms27198556/s1.

## Abbreviations

The following abbreviations are used in this manuscript:

PD	Parkinson’s disease
SNP	Single nucleotide polymorphism
RERI	Relative excess risk due to interaction
AP	Attributable proportion due to interaction
S	Synergy index
GWAS	Genome-wide association study
DAT	Dopamine transporter
NCBI	National Centre for Biotechnology Information

## References

[1] Jankovic J., Tan E.K. (2020). Parkinson’s Disease: Etiopathogenesis and Treatment. J. Neurol. Neurosurg. Psychiatry.

[2] Tan L.C.S., Venketasubramanian N., Hong C.Y., Sahadevan S., Chin J.J., Krishnamoorthy E.S., Tan A.K.Y., Saw S.M. (2004). Prevalence of Parkinson Disease in Singapore: Chinese vs Malays vs Indians. Neurology.

[3] Feigin V.L., Abajobir A.A., Abate K.H., Abd-Allah F., Abdulle A.M., Abera S.F., Abyu G.Y., Ahmed M.B., Aichour A.N., Aichour I. (2017). Global, Regional, and National Burden of Neurological Disorders during 1990–2015: A Systematic Analysis for the Global Burden of Disease Study 2015. Lancet Neurol..

[4] Ben-Shlomo Y., Darweesh S., Llibre-Guerra J., Marras C., San Luciano M., Tanner C. (2024). The Epidemiology of Parkinson’s Disease. Lancet.

[5] Nalls M.A., Blauwendraat C., Vallerga C.L., Heilbron K., Bandres-Ciga S., Chang D., Tan M., Kia D.A., Noyce A.J., Xue A. (2019). Identification of Novel Risk Loci, Causal Insights, and Heritable Risk for Parkinson’s Disease: A Meta-Analysis of Genome-Wide Association Studies. Lancet Neurol..

[6] Kim J.J., Vitale D., Otani D.V., Lian M.M., Heilbron K., Iwaki H., Lake J., Solsberg C.W., Leonard H., Makarious M.B. (2024). Multi-Ancestry Genome-Wide Association Meta-Analysis of Parkinson’s Disease. Nat. Genet..

[7] Foo J.N., Chew E.G.Y., Chung S.J., Peng R., Blauwendraat C., Nalls M.A., Mok K.Y., Satake W., Toda T., Chao Y. (2020). Identification of Risk Loci for Parkinson Disease in Asians and Comparison of Risk Between Asians and Europeans: A Genome-Wide Association Study. JAMA Neurol..

[8] Ascherio A., Schwarzschild M.A. (2016). The Epidemiology of Parkinson’s Disease: Risk Factors and Prevention. Lancet Neurol..

[9] Jacobs B.M., Belete D., Bestwick J., Blauwendraat C., Bandres-Ciga S., Heilbron K., Dobson R., Nalls M.A., Singleton A., Hardy J. (2020). Parkinson’s Disease Determinants, Prediction and Gene-Environment Interactions in the UK Biobank. J. Neurol. Neurosurg. Psychiatry.

[10] Bogers J.S., Bloem B.R., Den Heijer J.M. (2023). The Etiology of Parkinson’s Disease: New Perspectives from Gene-Environment Interactions. J. Park. Dis..

[11] McCulloch C.C., Kay D.M., Factor S.A., Samii A., Nutt J.G., Higgins D.S., Griffith A., Roberts J.W., Leis B.C., Montimurro J.S. (2008). Exploring Gene-Environment Interactions in Parkinson’s Disease. Hum. Genet..

[12] Gatto N.M., Rhodes S.L., Manthripragada A.D., Bronstein J., Cockburn M., Farrer M., Ritz B. (2010). A-Synuclein Gene May Interact with Environmental Factors in Increasing Risk of Parkinson’s Disease. Neuroepidemiology.

[13] Miyake Y., Tanaka K., Fukushima W., Kiyohara C., Sasaki S., Tsuboi Y., Oeda T., Shimada H., Kawamura N., Sakae N. (2016). *PARK16* Polymorphisms, Interaction with Smoking, and Sporadic Parkinson’s Disease in Japan. J. Neurol. Sci..

[14] Miyake Y., Tsuboi Y., Koyanagi M., Fujimoto T., Shirasawa S., Kiyohara C., Tanaka K., Fukushima W., Sasaki S., Yamada T. (2010). *LRRK2* Gly2385Arg Polymorphism, Cigarette Smoking, and Risk of Sporadic Parkinson’s Disease: A Case–Control Study in Japan. J. Neurol. Sci..

[15] Miyake Y., Tanaka K., Fukushima W., Kiyohara C., Sasaki S., Tsuboi Y., Yamada T., Oeda T., Shimada H., Kawamura N. (2012). SNCA Polymorphisms, Smoking, and Sporadic Parkinson’s Disease in Japanese. Park. Relat. Disord..

[16] Chen C., Zhu S., Zheng Z., Ding X., Shi W., Xia T., Gu X. (2025). A Genome-Wide Study on the Genetic and Causal Effects of Smoking in Neurodegeneration. J. Transl. Med..

[17] Gallo V., Vineis P., Cancellieri M., Chiodini P., Barker R.A., Brayne C., Pearce N., Vermeulen R., Panico S., Bueno-de-Mesquita B. (2019). Exploring Causality of the Association between Smoking and Parkinson’s Disease. Int. J. Epidemiol..

[18] Ritz B., Ascherio A., Checkoway H., Marder K.S., Nelson L.M., Rocca W.A., Ross G.W., Strickland D., Van Den Eeden S.K., Gorell J. (2007). Pooled Analysis of Tobacco Use and Risk of Parkinson Disease. Arch. Neurol..

[19] Mappin-Kasirer B., Pan H., Lewington S., Kizza J., Gray R., Clarke R., Peto R. (2020). Tobacco Smoking and the Risk of Parkinson Disease. Neurology.

[20] Tanner C.M., Goldman S.M., Aston D.A., Ottman R., Ellenberg J., Mayeux R., Langston J.W. (2002). Smoking and Parkinson’s Disease in Twins. Neurology.

[21] Vieira H.L.A., Queiroga C.S.F., Alves P.M. (2008). Pre-Conditioning Induced by Carbon Monoxide Provides Neuronal Protection against Apoptosis. J. Neurochem..

[22] Queiroga C.S.F., Vercelli A., Vieira H.L.A. (2015). Carbon Monoxide and the CNS: Challenges and Achievements. Br. J. Pharmacol..

[23] Jurado-Coronel J.C., Loaiza A.E., Díaz J.E., Cabezas R., Ashraf G.M., Sahebkar A., Echeverria V., González J., Barreto G.E. (2019). (E)-Nicotinaldehyde O-Cinnamyloxime, a Nicotine Analog, Attenuates Neuronal Cells Death Against Rotenone-Induced Neurotoxicity. Mol. Neurobiol..

[24] Cai Y., Zhang X., Zhou X., Wu X., Li Y., Yao J., Bai J. (2017). Nicotine Suppresses the Neurotoxicity by MPP+/MPTP through Activating α7nAChR/PI3K/Trx-1 and Suppressing ER Stress. NeuroToxicology.

[25] Pihlstrøm L., Rengmark A., Bjørnarå K.A., Dizdar N., Fardell C., Forsgren L., Holmberg B., Larsen J.P., Linder J., Nissbrandt H. (2015). Fine Mapping and Resequencing of the PARK16 Locus in Parkinson’s Disease. J. Hum. Genet..

[26] Satake W., Nakabayashi Y., Mizuta I., Hirota Y., Ito C., Kubo M., Kawaguchi T., Tsunoda T., Watanabe M., Takeda A. (2009). Genome-Wide Association Study Identifies Common Variants at Four Loci as Genetic Risk Factors for Parkinson’s Disease. Nat. Genet..

[27] Kolisek M., Montezano A.C., Sponder G., Anagnostopoulou A., Vormann J., Touyz R.M., Aschenbach J.R. (2015). PARK7/DJ-1 Dysregulation by Oxidative Stress Leads to Magnesium Deficiency: Implications in Degenerative and Chronic Diseases. Clin. Sci..

[28] Kolisek M., Nestler A., Vormann J., Schweigel-Röntgen M. (2012). Human Gene SLC41A1 Encodes for the Na+/Mg2+ Exchanger. Am. J. Physiol. Cell Physiol..

[29] Yang Y., Chen S., Zhang L., Zhang G., Liu Y., Li Y., Zou L., Meng L., Tian Y., Dai L. (2024). The PM20D1-NADA Pathway Protects against Parkinson’s Disease. Cell Death Differ..

[30] MacLeod D.A., Rhinn H., Kuwahara T., Zolin A., Di Paolo G., McCabe B.D., Marder K.S., Honig L.S., Clark L.N., Small S.A. (2013). *RAB7L1* Interacts with *LRRK2* to Modify Intraneuronal Protein Sorting and Parkinson’s Disease Risk. Neuron.

[31] Wu H., Eckhardt C.M., Baccarelli A.A. (2023). Molecular Mechanisms of Environmental Exposures and Human Disease. Nat. Rev. Genet..

[32] Sherry S.T., Ward M., Sirotkin K. (1999). dbSNP—Database for Single Nucleotide Polymorphisms and Other Classes of Minor Genetic Variation. Genome Res..

[33] Tan E.-K., Kwok H.-K., Tan L.C., Zhao W.-T., Prakash K.M., Au W.-L., Pavanni R., Ng Y.-Y., Satake W., Zhao Y. (2010). Analysis of GWAS-Linked Loci in Parkinson Disease Reaffirms PARK16 as a Susceptibility Locus. Neurology.

[34] Ahlbom A., Alfredsson L. (2005). Interaction: A Word with Two Meanings Creates Confusion. Eur. J. Epidemiol..

[35] Zhu H., Tonelli F., Turk M., Prescott A., Alessi D.R., Sun J. (2023). Rab29-Dependent Asymmetrical Activation of Leucine-Rich Repeat Kinase 2. Science.

[36] Kumari N., Cooke L.E., Olsen A.L. (2025). Proposed Mechanisms of Neuroprotection for Nicotine in Parkinson’s Disease. J. Park. Dis..

[37] Kume T., Takada-Takatori Y. (2018). Nicotinic Acetylcholine Receptor Signaling: Roles in Neuroprotection. Nicotinic Acetylcholine Receptor Signaling in Neuroprotection.

[38] Rojas-Rodríguez F., Morantes C., Pinzón A., Barreto G.E., Cabezas R., Mariño-Ramírez L., González J. (2020). Machine Learning Neuroprotective Strategy Reveals a Unique Set of Parkinson Therapeutic Nicotine Analogs. Open Bioinform. J..

[39] Oertel W.H., Müller H.-H., Unger M.M., Schade-Brittinger C., Balthasar K., Articus K., Brinkman M., Venuto C.S., Tracik F., Eberling J. (2023). Transdermal Nicotine Treatment and Progression of Early Parkinson’s Disease. NEJM Evid..

[40] Rose K.N., Zorlu M., Fassini A., Lee H., Cai W., Xue X., Lin S., Kivisakk P., Schwarzschild M.A., Chen X. (2024). Neuroprotection of Low Dose Carbon Monoxide in Parkinson’s Disease Models Commensurate with the Reduced Risk of Parkinson’s among Smokers. npj Park. Dis..

[41] Lim A.W.-Y., Foo J.-N., Chew E.G.-Y., Lim S.-Y., Tan A.H., Hwang L.-D., MacGregor S., Rentería M.E., Ong J.-S. (2026). Proteomic and Genetic Insights into Ancestry-Specific Associations in Parkinson’s Disease. Mov. Disord..

[42] Jończyk K., Kicman A., Michalska-Falkowska A., Śniadach J., Waszkiewicz N. (2025). Non-Invasive Exposure Biomarkers of Tobacco Smoke Exposure in Smokers of Classic Cigarettes and Users of e-Cigarettes and Heated Tobacco Products. Front. Mol. Biosci..

[43] Brody A.L., Olmstead R.E., London E.D., Farahi J., Meyer J.H., Grossman P., Lee G.S., Huang J., Hahn E.L., Mandelkern M.A. (2004). Smoking-Induced Ventral Striatum Dopamine Release. Am. J. Psychiatry.

[44] Wang C., Zhou C., Guo T., Huang P., Xu X., Zhang M. (2022). Association between Cigarette Smoking and Parkinson’s Disease: A Neuroimaging Study. Ther. Adv. Neurol. Disord..

[45] Gibb W.R., Lees A.J. (1988). The Relevance of the Lewy Body to the Pathogenesis of Idiopathic Parkinson’s Disease. J. Neurol. Neurosurg. Psychiatry.

[46] Hughes A.J., Daniel S.E., Ben-Shlomo Y., Lees A.J. (2002). The Accuracy of Diagnosis of Parkinsonian Syndromes in a Specialist Movement Disorder Service. Brain.

[47] Aune D., Schlesinger S., Mahamat-Saleh Y., Zheng B., Udeh-Momoh C.T., Middleton L.T. (2023). Diabetes Mellitus, Prediabetes and the Risk of Parkinson’s Disease: A Systematic Review and Meta-Analysis of 15 Cohort Studies with 29.9 Million Participants and 86,345 Cases. Eur. J. Epidemiol..

[48] Ng Y.-F., Ng E., Lim E.-W., Prakash K.M., Tan L.C.S., Tan E.-K. (2021). Case-Control Study of Hypertension and Parkinson’s Disease. npj Park. Dis..

[49] Hu G., Antikainen R., Jousilahti P., Kivipelto M., Tuomilehto J. (2008). Total Cholesterol and the Risk of Parkinson Disease. Neurology.

[50] Hurh K., Park M., Jang S., Park E.-C., Jang S.-Y. (2022). Association between Serum Lipid Levels over Time and Risk of Parkinson’s Disease. Sci. Rep..

[51] Yip C.-W., Tan E.-K. (2008). Test–Retest Repeatability of Assessing Environmental and Lifestyle Factors in Parkinson’s Disease. Mov. Disord..

[52] Gauderman W.J. (2002). Sample Size Requirements for Matched Case-Control Studies of Gene–Environment Interaction. Stat. Med..

[53] Andersson T., Alfredsson L., Källberg H., Zdravkovic S., Ahlbom A. (2005). Calculating Measures of Biological Interaction. Eur. J. Epidemiol..

