# Supplementary material for: Interaction Between Cigarette Smoking and PARK16 on Parkinson’s Disease
Source: Int J Mol Sci. 2026 Sep 24;27(19):8556. doi: 10.3390/ijms27198556 (PMC13665394; doi:10.3390/ijms27198556)
Supplement: Supplementary file 1 [file ijms-27-08556-s001.zip › ijms-4562376-supplementary.pdf]

Supplementary Table S1. Sensitivity analysis excluding covariates (hypertension, hyperlipidaemia, and diabetes mellitus) for interaction between *PARK16* rs6679073 and smoking status on risk of PD.

**Genetic model**	**Smoking status**	**Geno-type**	**n (%)**		Crude OR (95% CI)	Adjusted OR (95% CI) [1]	*P*-value
			**Controls**	**Cases**			
Recessive	Yes	CC+CA	63 (12.5)	55 (11.1)	1.00	1.00	
		AA	33 (6.6)	27 (5.4)	0.94 (0.50–1.75)	0.90 (0.47–1.70)	0.734
	No	CC+ CA	291 (58.0)	255 (51.3)	1.00 (0.67–1.50)	1.03 (0.67–1.59)	0.896
		AA	115 (22.9)	160 (32.2)	1.59 (1.03–2.46)	1.68 (1.06–2.68) [2]	0.029

[1] Logistic regression models were adjusted for age, sex, and family history.

[2] Interaction analyses: relative excess risk due to interaction (RERI) = 0.76 (95% CI 0.11–1.41); attributable proportion due to interaction (AP) = 0.45 (95% CI 0.058–0.84); Multiplicative interaction OR 1.83 (95% CI 0.90–3.70, *P*=0.095). Synergy index (S) was not estimable.
